# Proofreading-Deficient Coronaviruses Adapt for Increased Fitness over Long-Term Passage without Reversion of Exoribonuclease-Inactivating Mutations

**DOI:** 10.1128/mBio.01503-17

**Published:** 2017-11-07

**Authors:** Kevin W. Graepel, Xiaotao Lu, James Brett Case, Nicole R. Sexton, Everett Clinton Smith, Mark R. Denison

**Affiliations:** a Department of Pathology, Microbiology and Immunology, Vanderbilt University Medical Center, Nashville, Tennessee, USA; b Department of Pediatrics, Vanderbilt University Medical Center, Nashville, Tennessee, USA; c Elizabeth B. Lamb Center for Pediatric Research, Vanderbilt University Medical Center, Nashville, Tennessee, USA; d Department of Biology, the University of the South, Sewanee, Tennessee, USA; NIAID, NIH

**Keywords:** RNA virus, adaptive evolution, competitive fitness, coronavirus, exoribonuclease, plus-strand RNA virus, proofreading, replication fidelity

## Abstract

The coronavirus (CoV) RNA genome is the largest among the single-stranded positive-sense RNA viruses. CoVs encode a proofreading 3′-to-5′ exoribonuclease within nonstructural protein 14 (nsp14-ExoN) that is responsible for CoV high-fidelity replication. Alanine substitution of ExoN catalytic residues [ExoN(-)] in severe acute respiratory syndrome-associated coronavirus (SARS-CoV) and murine hepatitis virus (MHV) disrupts ExoN activity, yielding viable mutant viruses with defective replication, up to 20-fold-decreased fidelity, and increased susceptibility to nucleoside analogues. To test the stability of the ExoN(-) genotype and phenotype, we passaged MHV-ExoN(-) 250 times in cultured cells (P250), in parallel with wild-type MHV (WT-MHV). Compared to MHV-ExoN(-) P3, MHV-ExoN(-) P250 demonstrated enhanced replication and increased competitive fitness without reversion at the ExoN(-) active site. Furthermore, MHV-ExoN(-) P250 was less susceptible than MHV-ExoN(-) P3 to multiple nucleoside analogues, suggesting that MHV-ExoN(-) was under selection for increased replication fidelity. We subsequently identified novel amino acid changes within the RNA-dependent RNA polymerase and nsp14 of MHV-ExoN(-) P250 that partially accounted for the reduced susceptibility to nucleoside analogues. Our results suggest that increased replication fidelity is selected in ExoN(-) CoVs and that there may be a significant barrier to ExoN(-) reversion. These results also support the hypothesis that high-fidelity replication is linked to CoV fitness and indicate that multiple replicase proteins could compensate for ExoN functions during replication.

## IMPORTANCE

Uniquely among RNA viruses, CoVs encode a proofreading exoribonuclease (ExoN) in nsp14 that mediates high-fidelity RNA genome replication. Proofreading-deficient CoVs with disrupted ExoN activity [ExoN(-)] either are nonviable or have significant defects in replication, RNA synthesis, fidelity, fitness, and virulence. In this study, we showed that ExoN(-) murine hepatitis virus can adapt during long-term passage for increased replication and fitness without reverting the ExoN-inactivating mutations. Passage-adapted ExoN(-) mutants also demonstrate increasing resistance to nucleoside analogues that is explained only partially by secondary mutations in nsp12 and nsp14. These data suggest that enhanced resistance to nucleoside analogues is mediated by the interplay of multiple replicase proteins and support the proposed link between CoV fidelity and fitness.

## INTRODUCTION

A paradigm of RNA virus biology is error-prone genomic replication due to the lack of proofreading or postreplicative RNA repair mechanisms (1–3). Decreased replication fidelity may constrain RNA genome size and complexity and risks the accumulation of deleterious mutations leading to population extinction (4–7). While genetic diversity allows viral populations to adapt rapidly under selective pressure, many mutations are neutral or detrimental to viral fitness (8–12). Research performed with many RNA viruses supports the hypothesis that the mutation rate of RNA virus replicases has evolved to balance multiple characteristics of the viral population such as genetic diversity, genomic integrity, and virulence. High- or low-fidelity variants are described for many RNA viruses infecting animals, including the coronaviruses (CoVs) murine hepatitis virus (MHV-A59) and severe acute respiratory syndrome-associated coronavirus (SARS-CoV) (13–17), as well as foot-and-mouth disease virus (18–22), poliovirus (23–29), Chikungunya virus (30, 31), influenza virus (32), coxsackievirus B3 (33, 34), and human enterovirus 71 (35–37). Most altered-fidelity variants described to date harbor mutations within the viral RNA-dependent RNA polymerase (RdRp), are attenuated *in vivo*, and protect against reinfection, highlighting their potential utility as live attenuated vaccines (24, 28, 29, 38, 39). Those studies underscored the importance of understanding the molecular mechanisms by which RNA viruses regulate their replication fidelity.

**FIG 1:**
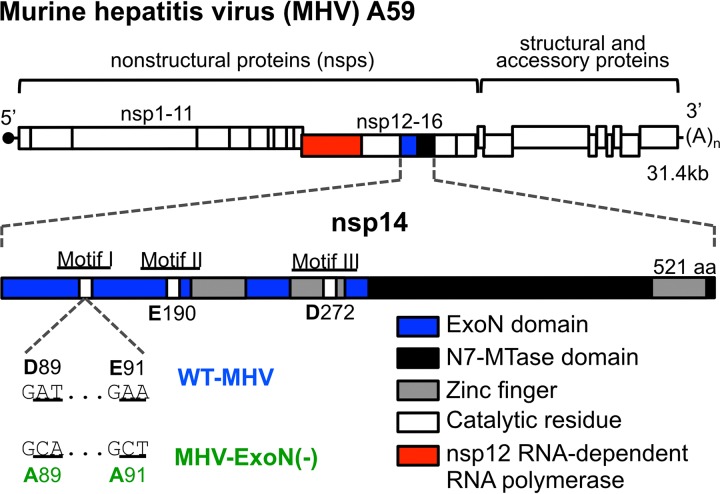
MHV genome organization and nsp14 exoribonuclease motifs. (Top) The MHV genome is a 31.4-kb, capped (dark circle), and polyadenylated positive-sense RNA molecule. The first two-thirds of the genome encode 16 nonstructural proteins translated as a single polyprotein with a ribosomal frameshift. The final one-third encodes the structural and accessory proteins. (Inset) Nsp14 encodes an exoribonuclease (solid blue) and an N7-methyltransferase (hatched blue) and has 3 zinc fingers (gray boxes) predicted from the solved SARS nsp10/14 crystal structure (PDB 5C8U) (44). Catalytic residues for ExoN are marked with white boxes, and the engineered mutations for MHV-ExoN(-) are shown below the genome. The nsp12 RNA-dependent RNA polymerase is highlighted in red.

Viruses in the *Coronavirinae* subfamily have large single-stranded positive-sense RNA genomes [(+)ssRNA] (40), ranging between 26 and 32 kb in length (41). CoVs encode a 3′-to-5′ exoribonuclease (ExoN) in the N-terminal half of nonstructural protein 14 (nsp14-ExoN) (42, 43). CoV ExoN activity depends on conserved magnesium-coordinating acidic amino acids in three motifs (DE-E-D) that together constitute the active site (Fig. 1) (44). The CoV ExoN is grouped with the DE-D-Dh superfamily of exonucleases involved in proofreading during prokaryotic and eukaryotic DNA replication (42–46). Alanine substitution of CoV motif I DE residues (DE-to-AA) reduces biochemical ExoN activity in SARS-CoV (44, 46) and human coronavirus 229E (42). MHV-A59 and SARS-CoV lacking ExoN activity [ExoN(-)] have mutation frequencies 8-fold to 20-fold greater than are seen with WT viruses and are highly susceptible to the activity of nucleoside analogues (13–17, 38). Thus, all available data to date support the hypothesis that nsp14-ExoN is the first known proofreading enzyme encoded by an RNA virus.

Despite the critical role of ExoN in virus replication, fidelity, fitness, and virulence, reversion of the ExoN-inactiviting substitutions (Fig. 1) has not been detected following 20 passages in culture, 8 acute passages of SARS-CoV-ExoN(-) in aged BALB/c mice, and 60 days of persistent SARS-CoV-ExoN(-) infection in immunodeficient Rag^−/−^ mice (13, 14, 16, 17, 38). In this study, we sought to determine whether long-term passage of MHV-A59-ExoN(-) (250 passages over 1 year [P250])—here MHV-ExoN(-)—would result in virus extinction, ExoN(-) reversion, or compensation for the loss of proofreading. We demonstrate that MHV-ExoN(-) did not extinguish during passage and adapted for increased replication. MHV-ExoN(-) concurrently evolved reduced susceptibility to multiple nucleoside and base analogues, consistent with selection for increased replication fidelity. Importantly, the ExoN-inactivating substitutions did not revert. The evolved mutations in MHV-ExoN(-) nsp14 and nsp12, which encodes the RdRp, accounted for only part of the increased nucleoside analogue resistance of MHV-ExoN(-) P250, implicating multiple replicase proteins in adaptation for viral fitness. The results of this study support the proposed link between CoV fidelity and fitness, demonstrate the surprising stability of the ExoN-inactivating substitutions, and identify additional proteins outside nsp12 and nsp14 that may contribute to CoV fidelity regulation.

## RESULTS

### Long-term passage of WT-MHV and MHV-ExoN(-).

We serially passaged WT-MHV and MHV-ExoN(-) in delayed brain tumor (DBT) cells 250 times (P250). Virus from each passage was harvested once 50% to 100% of the monolayer was involved in syncytia, which occurred between 8 and 24 hours postinfection (hpi). Passage conditions varied for WT-MHV and MHV-ExoN(-) due to differences in replication kinetics between the two viruses. We stopped passage at P250 after observing reduced syncytium formation in MHV-ExoN(-)-infected flasks, likely resulting from a mutation in the MHV-ExoN(-) P250 spike protein cleavage site (discussed below).

### MHV-ExoN(-) and WT-MHV replicate with identical kinetics following 250 passages.

**FIG 2:**
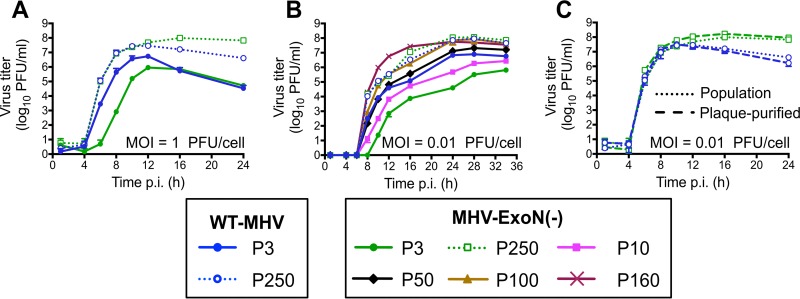
MHV-ExoN(-) evolved increased replicative capacity over long-term passage. Replication kinetics were examined for the indicated viruses at MOI = 1 PFU/cell (A) and MOI = 0.01 PFU/cell (B). (C) Replication kinetics of plaque-purified WT-MHV P250 and MHV-ExoN(-) P250 in parallel with the full population (MOI = 0.01 PFU/cell). Supernatants were collected at the indicated times postinfection, and titers were determined by plaque assay. Data for panels A to C represent means and standard deviations of data from *n* = 3.

MHV-ExoN(-) has a significant replication defect relative to WT-MHV (14). We first tested whether replication of MHV-ExoN(-) P250 was affected by long-term passage by examining replication at two different multiplicities of infection (MOI). At both MOI = 1 and MOI = 0.01 PFU/cell, MHV-ExoN(-) P3 replication was delayed by ~2 h and the peak titer was reduced by ~1 log_10_ relative to WT-MHV P3 (Fig. 2A and B), consistent with our previous studies (14). By P250, the two viruses replicated with identical kinetics (Fig. 2A and B, dotted lines). This represented an ~1 log_10_ increase in peak replication for WT-MHV and an ~2 log_10_ increase for MHV-ExoN(-), compared with the respective parental viruses. At MOI = 0.01 PFU/cell, we also measured replication of MHV-ExoN(-) at P10, P50, P100, and P160. Replication kinetics gradually increased during the passages, reaching P250-like levels by P100 (Fig. 2B). To determine whether the increased replication of MHV-ExoN(-) P250 was affected by the presence of potential defective viral genomes or by some other population-based phenomenon, both WT-MHV P250 and MHV-ExoN(-) P250 were plaque purified three times. The plaque-purified viruses replicated indistinguishably from the parent populations (Fig. 2C). Together, these data demonstrate that WT-MHV and MHV-ExoN(-) populations had adapted for increased replication and that either individual genomes or those derived from a single virus plaque encoded the adaptive changes required by the total population.

### MHV-ExoN(-) accumulated 8-fold-more mutations than WT-MHV but did not revert ExoN-inactivating substitutions.

**FIG 3:**
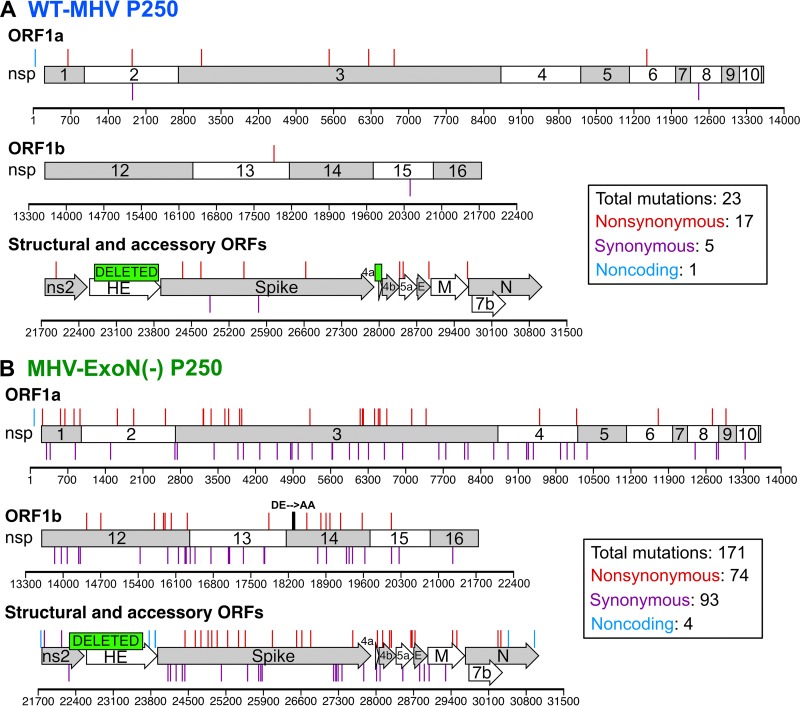
Mutations within P250 viruses. The mutations shown were present at >50% by di-deoxy sequencing at passage 250 in WT-MHV (A) and MHV-ExoN(-) (B). Nonsynonymous mutations (red), noncoding mutations (cyan), and deletions (green boxes) are plotted above the schematic, and synonymous mutations (purple) are plotted below the schematic.

To determine whether the increased replication of MHV-ExoN(-) P250 resulted from primary reversion of ExoN(-) motif I, we sequenced nsp14 from infected-cell total RNA. MHV-ExoN(-) P250 retained the motif I DE-to-AA substitutions, demonstrating that primary reversion of ExoN(-) motif I did not occur. To identify potentially adaptive consensus mutations, we performed full-genome di-deoxy sequencing of MHV-ExoN(-) P250 and WT-MHV P250. Within WT-MHV P250, we identified 23 mutations, of which 17 were nonsynonymous (NS) (Fig. 3A). In contrast, MHV-ExoN(-) P250 had 171 total mutations (74 NS) (Fig. 3B). The full-genome sequences have been deposited in GenBank, and the mutations for both viruses are listed in Tables S1 and S2 in the supplemental material. We identified only one mutation shared by both viruses (nsp1 A146T), though it was present in approximately 50% of the WT-MHV P250 population by di-deoxy sequencing. Both viruses deleted most of the hemagglutinin esterase (HE). In MHV-A59, HE mRNA is not transcribed *in vitro* (47–49), and HE protein expression is detrimental to MHV-A59 fitness in cell culture (50). WT-MHV P250 also deleted open reading frame 4a (ORF4a), which is dispensable for MHV replication in cell culture (51). The C-terminal region of ns2 within MHV-ExoN(-) P250 was truncated and fused to HE with a −1 frameshift. Ns2 is a phosphodiesterase (PDE) that protects viral RNA by degrading 2′-to-5′ oligoadenylate, the activating factor for cellular RNase L (52–54). The portion of ns2 deleted in MHV-ExoN(-) P250 lies outside the PDE catalytic domain, in a region of unknown function. C-terminally truncated ns2 retains enzymatic activity (55), but whether these specific deletions and fusions disrupt PDE activity remains to be tested. Nevertheless, ns2 is dispensable for MHV replication in immortalized cells (56, 57). Details about the deletion sites are provided in Fig. S1 in the supplemental material. Within proteins predicted to be part of the replicase-transcriptase complex (nsp7-16 and nucleocapsid) (39), WT-MHV P250 had only one NS change, located in the nsp13-helicase (Fig. 3A and Table S1). In contrast, MHV-ExoN(-) P250 had 17 NS changes within this region (Fig. 3B and Table S2).

10.1128/mBio.01503-17.2 TABLE S1  Mutations in WT-MHV P250. Download TABLE S1, PDF file, 0.1 MB. Copyright © 2017 Graepel et al. 2017 Graepel et al. This content is distributed under the terms of the Creative Commons Attribution 4.0 International license.

10.1128/mBio.01503-17.3 TABLE S2  Mutations in MHV-ExoN(-) P250. Download TABLE S2, PDF file, 0.1 MB. Copyright © 2017 Graepel et al. 2017 Graepel et al. This content is distributed under the terms of the Creative Commons Attribution 4.0 International license.

10.1128/mBio.01503-17.1 FIG S1  Deleted regions within WT-MHV P250 and MHV-ExoN(-) P250. Download FIG S1, TIF file, 1.7 MB. Copyright © 2017 Graepel et al. 2017 Graepel et al. This content is distributed under the terms of the Creative Commons Attribution 4.0 International license.

### MHV-ExoN(-) P250 displays increased genomic RNA accumulation and increased resistance to 5-fluorouracil.

**FIG 4:**
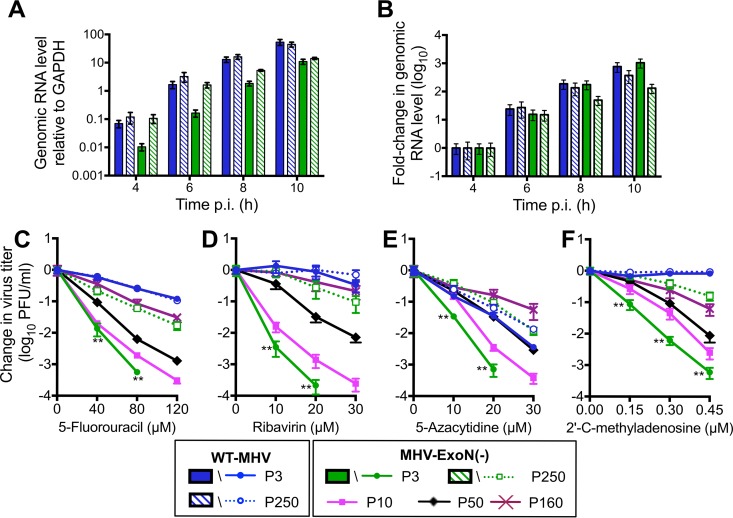
MHV-ExoN(-) evolved WT-like genomic RNA accumulation and increased resistance to multiple nucleoside analogues over the passage. (A) Cells were infected with the indicated viruses at MOI = 1 PFU/cell, and intracellular RNA was harvested using TRIzol at the indicated times postinfection. MHV genomic RNA was detected using SYBR green and primers directed to nsp10, and values were normalized to intracellular GAPDH. (B) Same data as in panel A normalized to the RNA level for each virus at 4 hpi. Data represent means and standard errors of results for *n* = 9 (3 triplicate experiments). (C to F) Sensitivity of passaged viruses to nucleoside analogues at MOI = 0.01 PFU/cell. Cells were treated with the indicated concentrations of 5-FU (C), RBV (D), AZC (E), or CMeA (F) for 30 min prior to infection, supernatants were harvested at 24 hpi, and titers were determined by plaque assay. Data represent changes in titer relative to untreated control results and are plotted as means and standard errors of results from *n* = 6 (two triplicate experiments). For panels C to F, the statistical significance of changes in the titer of MHV-ExoN(-) P3 relative to MHV-ExoN(-) P250 was determined using the Mann-Whitney test (*, *P* < 0.05; **, *P* < 0.01; ***, *P* < 0.001).

Coronaviruses lacking ExoN consistently display defects in RNA synthesis relative to WT strains (14, 16, 42). To determine whether the increased replication of MHV-ExoN(-) P250 was associated with restored genomic RNA (gRNA) production, we measured gRNA accumulation over time using two-step real-time quantitative PCR (15, 16). MHV-ExoN(-) P250 accumulated levels of gRNA similar to those accumulated by WT-MHV P3 and WT-MHV P250 at early time points, while gRNA levels for MHV-ExoN(-) P3 were ~1 log_10_ lower (Fig. 4A). MHV-ExoN(-) P250 gRNA levels fell below those of WT-MHV and WT-MHV P250 after 8 h and were similar to those of MHV-ExoN(-) P3 at 10 hpi. Normalizing to the gRNA abundance at 4 h for each virus demonstrated that the rates of gRNA accumulation were similar for all four viruses (Fig. 4B). These data suggest that the increased replication of P250 viruses relative to WT-MHV is not fully accounted for by increased RNA synthesis. In addition to RNA synthesis defects, ExoN(-) CoVs have up to 20-fold-increased mutation frequencies and profoundly increased sensitivity to nucleoside and base analogues relative to WT CoVs (13, 14, 16, 17, 38). To determine whether the nucleoside analogue sensitivity of MHV-ExoN(-) was altered by long-term passage, we treated cells infected with parental and passaged viruses with the base analog, 5-fluorouracil (5-FU). 5-FU is converted intracellularly into a nucleoside analogue that incorporates into growing RNA strands and causes A:G and U:C mutations. For simplicity, we will hereafter refer to 5-FU as a nucleoside analogue. Incorporation of 5-FU is increased in the absence of ExoN activity (16). All viruses displayed a concentration-dependent decrease in viral titer but differed greatly in their levels of susceptibility to 5-FU (Fig. 4C). At 120 μM, WT-MHV P3 titers were reduced by ~1 log_10_, while MHV-ExoN(-) P3 titers were undetectable (>5 log_10_-fold reduction). WT-MHV 5-FU sensitivity was not altered by passage. MHV-ExoN(-) P250 was less susceptible than MHV-ExoN(-) P3 to 5-FU treatment, with a decrease in titer of only ~1.5 log_10_ at 120 μM. MHV-ExoN(-) P250 remained more sensitive to 5-FU than WT-MHV, suggesting that WT-like resistance requires an intact ExoN. These data demonstrate that MHV-ExoN(-) P3 evolved resistance to 5-FU through mutations outside ExoN(-) motif I.

### Spike mutations in MHV-ExoN(-) P250 do not increase resistance to 5-FU.

**FIG 5:**
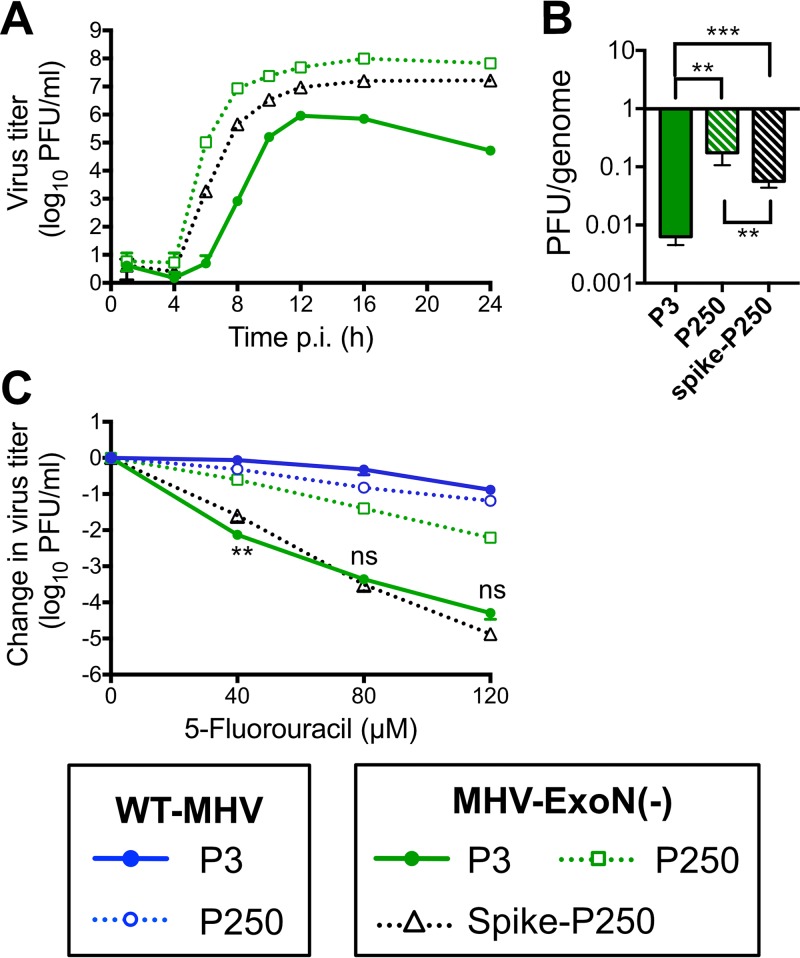
Mutations in the spike envelope protein from MHV-ExoN(-) P250 increase replicative capacity but do not affect sensitivity to 5-fluorouracil. (A) Replication kinetics of indicated viruses (MOI = 0.01 PFU/cell) plotted as means and standard deviations of results determined with *n* = 3. (B) Specific infectivity of indicated viruses 12 hpi (MOI = 1 PFU/cell). Data represent means and standard errors of results from *n* = 6 (two triplicate experiments). (C) Sensitivity of indicated viruses to 5-fluorouracil at MOI = 0.01 PFU/cell, determined as described for Fig. 4. Data represent means and standard errors of results from *n* = 6 (two triplicate experiments). For panel B, the statistical significance was determined using one-way analysis of variance (ANOVA). For panel C, the statistical significance of changes in the titer of MHV-ExoN(-) spike-P250 relative to MHV-ExoN(-) P3 was determined using the Mann-Whitney test (*, *P* < 0.05; **, *P* < 0.01; ***, *P* < 0.001; ns, not significant).

Bacteriophage ϕX174 acquired resistance to 5-FU by delaying cell lysis, thereby reducing the number of replication cycles in which 5-FU can be incorporated (58). MHV-ExoN(-) P250 had multiple mutations in the spike glycoprotein, including one in the spike furin cleavage site that reduced syncytium formation. To test whether the spike mutations manifested in resistance to 5-FU, we cloned the spike gene from MHV-ExoN(-) P250 into the isogenic MHV-ExoN(-) background. The recombinant virus demonstrated intermediate replication kinetics between MHV-ExoN(-) P3 and MHV-ExoN(-) P250 (Fig. 5A) and did not form syncytia. Spike-P250 also increased the specific infectivity of viral particles (Fig. 5B). However, the MHV-ExoN(-) P250 spike did not affect the sensitivity of the recombinant virus to 5-FU (Fig. 5C). Thus, any adaptive increase in 5-FU resistance must be located elsewhere in the genome.

### MHV-ExoN(-) passage resulted in unique mutations in nsp12 and nsp14.

**FIG 6:**
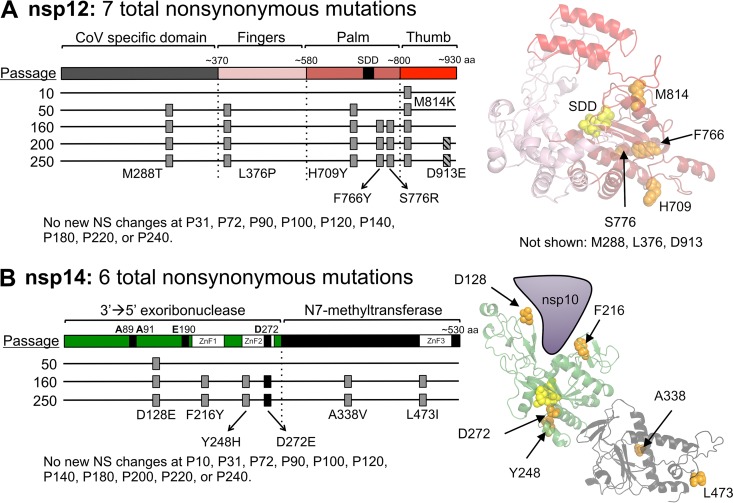
The timing of fixation of mutations in nsp12-RdRp and nsp14-ExoN within MHV-ExoN(-). (A) A schematic of nsp12-RdRp with the CoV-specific region and the canonical finger, palm, and thumb domains of RdRps is shown. The nsp12-RdRp coding region was sequenced at the indicated passage, and the nonsynonymous changes are plotted; gray boxes indicate consensus changes, and hatched boxes indicate variants shown to be present in <50% of the population by di-deoxy sequencing. At right, mutations are marked in orange on a Phyre^2^-modeled structure of MHV-nsp12, with the active site residues marked in yellow (17). RdRp domains are colored according to the linear schematic. M288T, L376P, and D913E lie outside the modeled region and thus are not marked. (B) A schematic of nsp14 with the ExoN and N7-methyltransferase domains is shown, with mutation plotting depicted as described for panel A. The black box denotes a mutation to ExoN motif III. At right, mutations are marked in orange on a Phyre^2^-modeled structure of MHV-nsp14. Domains are colored according to the linear schematic.

To date, three proteins have been shown to alter CoV sensitivity to 5-FU: nsp12-RdRp, nsp14-ExoN, and nsp10 (which stimulates ExoN activity) (15, 17, 39). Neither WT-MHV nor MHV-ExoN(-) P250 contained an NS mutation in nsp10, and WT-MHV P250 had no mutations within either nsp12 or nsp14. In contrast, MHV-ExoN(-) P250 had 7 NS mutations in nsp12 and 6 NS mutations in nsp14 (Fig. 3 and 6), none of which have been described previously *in vitro* or in viable viruses. Within nsp12, six mutations were in the predicted RdRp finger, palm, and thumb domains (Fig. 6A) (59). Four residues (H709, F766, S776, and M814) can be visualized on a Phyre^2^-modeled structure of the MHV-nsp12 RdRp, while the remaining residues lie outside the modeled core RdRp (Fig. 6A) (17). One mutation, M288T, lies in the CoV-specific domain, which is conserved among nidoviruses. This domain has been implicated in membrane targeting in MHV-A59 (60) and performs an essential nucleotidylation activity in the *Arterivirus* equine arteritis virus (61). However, M288T is not predicted to catalyze nucleotidylation. Within nsp14, 4 NS mutations were identified in the ExoN domain, and 2 NS mutations were in the C-terminal N7-methyltransferase domain (Fig. 6B). We next modeled the structure of MHV nsp14 using Phyre^2^ software (62), resulting in highest-probability similarity to the SARS-CoV nsp14-nsp10 complex (PDB 5C8S) (44) with high confidence (i.e., the calculated probability of true homology between the structures) of 100% for residues 3 to 519 of MHV-nsp14. The model predicts that five mutations are located close to surface of the protein (Fig. 6B). All three modeled zinc finger domains contain one NS mutation (F216Y, Y248H, and L473I). Two mutations, D128E and F216Y, are located near the interface between nsp10 and nsp14, though neither site has previously been implicated in nsp10-nsp14 interactions (15, 63, 64). One NS mutation resulted in a D272E substitution in ExoN motif III, a metal-coordinating active site residue. We previously reported that alanine substitution of D272 results in an ExoN(-) phenotype (14), but the viability or phenotype of a D272E substitution was not tested in that study. These data suggest that a network of residues evolved to regulate nsp12 and nsp14 activity or stability in the ExoN(-) background.

### Fixed mutations in nsp12 and nsp14 in MHV-ExoN(-) P250 directly correlate with increased resistance to multiple nucleoside analogues.

To determine approximately when the mutations in nsp12 and nsp14 arose, we performed di-deoxy sequencing across these protein-coding regions roughly every 20 passages (P10, P31, P50, P72, P90, P100, P120, P140, P160, P180, P200, P220, and 240). By this method, we detected consensus NS mutations at P10, P50, and P160 for nsp12 and at P50 and P160 for nsp14 (Fig. 6). Both nsp12 and nsp14 carried their full complement of P250 consensus mutations by P160, except for a minority variant (D913E) in nsp12 that was maintained at <50% of the population between P200 and P250. These passage levels correlated with increased replication of MHV-ExoN(-) (Fig. 2B) and with decreasing sensitivity to 5-FU (Fig. 4C). Neither replication nor 5-FU sensitivity of MHV-ExoN(-) changed substantially between P160 and P250. To determine whether MHV-ExoN(-) evolved increased resistance to multiple nucleoside analogues, we treated virus-infected cells with three additional analogues that are substrates for viral RdRps: ribavirin (RBV), a guanine analogue that inhibits viral replication through multiple mechanisms, including mutagenesis and inhibition of purine biosynthesis (65); 5-azacytidine (AZC), an RNA mutagen (66); and 2′-C-methyladenosine (CMeA), which is proposed to incorporate in viral RNA and terminate nascent transcripts (67). As with 5-FU, we observed dose-dependent sensitivity to RBV, AZC, and CMeA in all MHV-ExoN(-) viruses that decreased with increasing passage number (Fig. 4D to F). Except for AZC, MHV-ExoN(-) sensitivity did not change between P160 and P250. Together, these data demonstrate that MHV-ExoN(-) evolved increased resistance to multiple nucleoside analogues that correlated with the length of passage and the acquisition of mutations in nsp12 and nsp14. Importantly, this occurred in the absence of specific mutagenic selection and without reversion of ExoN motif I. This increased general selectivity toward all four classes of nucleotide strongly supports the idea of an overall increase in fidelity in MHV-ExoN(-) P250.

### Mutations in nsp12 partially account for increased resistance of MHV-ExoN(-) P250 to multiple nucleoside analogues.

**FIG 7:**
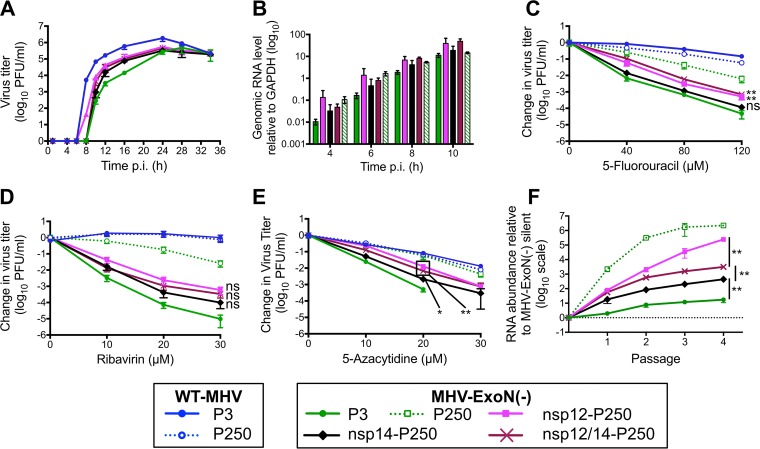
Mutations in nsp12-RdRp and nsp14-ExoN from MHV-ExoN(-) P250 incompletely increase resistance to nucleoside analogues and increase fitness of MHV-ExoN(-). (A) Replication kinetics of recombinant P250 viruses (MOI = 0.01 PFU/cell) plotted as means and standard deviations of results determined with *n* = 3. (B) Genomic RNA accumulation relative to intracellular GAPDH determined as described for Fig. 4. Data represent means and standard errors of results for *n* = 6 to 9 (2 to 3 triplicate experiments). (C to E) Sensitivity of recombinant MHV-ExoN(-) viruses to 5-FU (C), ribavirin (D), and 5-azacytidine (E) at MOI = 0.01 PFU/cell determined as described for Fig. 4. Data represent means and standard errors of results from *n* = 6. (F) Recombinant viruses were competed against a reference MHV-ExoN(-) containing 10 silent mutations within nsp2. The ratio of competitor to reference genomes is plotted. Data represent means and standard errors of results from *n* = 6. The MHV-ExoN(-) P250 data set contained 4 replicates at passage 3 and a single replicate at passage 4 due to undetectable levels of MHV-ExoN(-) (silent). For panels C to E, the statistical significance of changes in the titer of swapped viruses relative to MHV-ExoN(-) P3 at the highest drug concentration tolerated was determined using the Mann-Whitney test (*, *P* < 0.05; **, *P* < 0.01; ***, *P* < 0.001; ns, not significant). For panel F, the statistical significance for the indicated comparisons was determined using the Mann-Whitney test. Boxed points have the same *P* value.

We hypothesized that mutations in MHV-ExoN(-) P250 nsp12 and nsp14 were most likely to impact replication and nucleoside analogue sensitivity based on their enzymatic activities and temporal association with phenotypic changes. To test this hypothesis, we engineered recombinant MHV-ExoN(-) to encode the P250 nsp12 and nsp14 sequences, alone and together. Expression of nsp12-P250 and nsp14-P250, alone or in combination, altered replication kinetics of MHV-ExoN(-) without affecting peak titers (Fig. 7A) and increased gRNA levels above those of MHV-ExoN(-) P3 (Fig. 7B). Nsp12-P250 had a greater effect than nsp14-P250 on the sensitivity of MHV-ExoN(-) to all analogues tested, and the combination of nsp12- and nsp14-P250 did not increase resistance above that seen with nsp12-P250 alone (Fig. 7C to E). None of the recombinant viruses recapitulated the resistance phenotypes of the MHV-ExoN(-) P250 population. Together, these data demonstrate that nsp12-P250 mutations account only partially for the nucleoside analogue resistance of MHV-ExoN(-) P250 and that adaptations in nsp12-P250 mask those in nsp14-P250. We also can conclude that the nsp14-P250 D272E active site mutation does not correct the defect caused by the motif I DE-to-AA substitutions.

### Resistance to nucleoside analogues correlates with MHV-ExoN(-) fitness.

We hypothesized that mutations in nsp12 and nsp14 provided a fitness advantage to MHV-ExoN(-) P250. We competed the recombinant viruses with a reference MHV-ExoN(-) virus (P1 stock) containing 10 silent mutations in the nsp2 coding region. Mutant and reference viruses were detected in the mixed infection by real-time quantitative PCR using dually labeled probes specific for each virus. MHV-ExoN(-) P3 showed a modest fitness advantage over the reference P1 MHV-ExoN(-) silent strain (Fig. 7F, solid green). MHV-ExoN(-) P250 profoundly outcompeted MHV-ExoN(-) silent, with >1,000-fold more MHV-ExoN(-) P250 genomes present at the end of passage 1 (Fig. 7F, dotted green line). MHV-ExoN(-) nsp12-P250 had greater relative fitness than MHV-ExoN(-) nsp14-P250, and MHV-ExoN(-) nsp12/14-P250 was intermediate between the single recombinants, implicating a complex evolutionary interaction between these two proteins. The measured fitness correlated with the patterns of nucleoside analogue resistance and RNA synthesis associated with mutations in nsp12 and nsp14, suggesting a link between the evolutions of these phenotypes. The result also confirms that nsp12 and nsp14 are important but not sufficient to account for the significantly increased fitness of MHV-ExoN(-) P250 relative to MHV-ExoN(-) P3.

## DISCUSSION

In this report, we describe experimental adaptive evolution of WT-MHV and MHV-ExoN(-) during long-term passage in cell culture. WT-MHV evolved increased replication kinetics over 250 passages, with few consensus mutations arising in the WT-MHV P250 genome. In contrast, MHV-ExoN(-) accumulated 8-fold-more mutations than WT-MHV, none of which occurred at the ExoN-inactivating substitutions. Nevertheless, MHV-ExoN(-) P250 demonstrated increased replication kinetics and fitness compared to MHV-ExoN(-) P3 (Fig. 2 and 7). Our previous studies demonstrated that ExoN-mediated proofreading is required for CoV fitness (16, 17, 38). Thus, MHV-ExoN(-) was likely under selective pressure for restoration of high-fidelity replication or for tolerance of the increased mutational load. Consistent with this hypothesis, MHV-ExoN(-) P250 exhibited increased resistance to multiple nucleoside analogues, a phenotype strongly associated with high-fidelity viruses (15, 18, 19, 22). Our results raise several important questions. In the face of selection for increased fidelity, why did MHV-ExoN(-) not revert? Can MHV replicase proteins mediate high-fidelity replication without ExoN proofreading? Which mechanisms other than increased fidelity can compensate for the loss of proofreading?

### In the face of selective pressure for increased fidelity, why did MHV-ExoN(-) not revert?

Although our data suggest that MHV-ExoN(-) was under selective pressure for increased fidelity, we detected no primary reversion at the DE-to-AA substitutions in MHV-ExoN(-) at any passage tested. These data are consistent with and significantly extend previous studies reporting genotypic stability of ExoN(-) motif I in MHV and SARS-CoV (13, 14, 16, 17, 38). Complete reversion to DE within ExoN(-) motif I would require four nucleotide changes. This likely represents a high genetic barrier to reversion, especially given that fitness can be increased by mutations outside nsp14-ExoN (Fig. 7F) (13). Single and double nucleotide changes within motif I could restore an acidic charge to individual residues (e.g., motif I EA, AD, ED, etc.). However, the active site compositions of DEDDh exonucleases, such as the Klenow fragment, are so stringent that even conservative mutations (D to E or E to D) reduce ExoN activity by >96% (68). Thus, intermediate amino acid changes may not have a selective advantage compared to motif I AA, limiting the evolutionary pathways to reversion. However, nsp14-P250 had detectable effects on RBV and AZC resistance as well as on the competitive fitness of MHV-ExoN(-) (Fig. 7F), demonstrating a modest capacity for fitness adaptation in nsp14 outside the catalytic residues. Whether these mutations resulted from genetic drift or positive selection remains unclear. Nevertheless, our data show that MHV-ExoN(-) can adapt for increased fitness without fully restoring exoribonuclease activity. While some mutations in MHV-ExoN(-) P250 likely confer DBT cell-specific selective advantages, others may represent generalizable strategies for overcoming ExoN(-) defects in other cell types and in other coronaviruses. Thus, understanding the mechanisms by which MHV-ExoN(-) P250 compensated for ExoN activity could allow recovery of ExoN(-) variants of other CoVs, such as transmissible gastroenteritis virus and human CoV 229E, which to date have been nonviable as ExoN(-) recombinants (42, 69).

### Can MHV replicase proteins mediate high-fidelity replication without ExoN proofreading?

MHV-ExoN(-) P250 exhibits increased resistance to four nucleoside analogues after passage (Fig. 4). Although resistance to a single nucleoside analogue can evolve without increasing overall fidelity, resistance to multiple nucleoside analogues strongly suggests a broadly increased capacity to discriminate nucleotides (15, 18, 19, 22). Increased-fidelity variants in RNA viruses have most frequently been mapped to RdRps (24, 25, 30, 70). Thus, if increased fidelity contributes to nucleoside analogue resistance in MHV-ExoN(-) P250, the most likely protein involved would be nsp12-P250. Three findings are consistent with the hypothesis that mutations within nsp12-P250 increase RdRp fidelity. First, nonsynonymous mutations to nsp12 arose in the low-fidelity MHV-ExoN(-) strain but not in the presence of proofreading (WT-MHV). Second, five of the mutations lie in or near structural motifs important for fidelity regulation in other RdRps. Amino acid substitutions in the finger and palm domains have been repeatedly shown to affect viral RdRp fidelity (25, 34), and we have recently reported a finger mutation (nsp12-V553I) that likely increases the fidelity of the MHV RdRp (17). Our modeled structure predicts that nsp12-P250 contains three mutations in the palm domain and one in the finger domain, with the M814K thumb domain mutation lying near the palm (Fig. 6A). Third, exchange of nsp12-P250 alone into the background of MHV-ExoN(-) reduced the susceptibility of MHV-ExoN(-) to three different nucleoside analogues (Fig. 7). Thus, all data support the hypothesis that nsp12-P250 is a high-fidelity RdRp. We are actively developing biochemical, phenotypic, and deep sequencing assays to quantify the fidelity of nsp12-P250.

Importantly, nsp12-P250 accounts only partially for the MHV-ExoN(-) P250 nucleoside analogue resistance phenotype (Fig. 7), suggesting a possible limit to the compensation achievable by mutating the RdRp alone. Further, the effects of mutations in nsp12-P250 and nsp14-P250 are not additive and may be antagonistic when they are isolated from the whole passaged virus (Fig. 7), indicating that the relationships between nsp12- and nsp14-P250 mutations are likely evolutionarily linked with those in other MHV proteins. In fact, a substantial component of the evolved resistance to nucleoside analogues cannot be explained by the presence of nsp12-P250 and nsp14-P250, alone or together. In support of this hypothesis, we identified several nonsynonymous mutations in other replicase proteins, such as nsp8, nsp9, nsp13, and nsp15. SARS-CoV nsp8 and nsp13 have functional interactions with nsp12, acting as a primase/processivity factor (71, 72) and a helicase/NTPase, respectively (73). Processivity factors in herpes simplex virus and *Mycobacterium tuberculosis* regulate DNA polymerase fidelity by balancing polymerase extension and exonuclease activity (74, 75), and helicases in Chikungunya virus and foot-and-mouth disease virus can evolve to increase fidelity (76) and alter the frequency of ribavirin-induced mutations (77), respectively. SARS-CoV nsp9 has RNA-binding activities and is proposed to participate in the multiprotein replicase complex (39, 78), and MHV nsp15 is a uridylate-specific endoribonuclease (79). Both could plausibly be involved in modulating polymerase activity. Additionally, it remains possible that evolution for increased fidelity could involve proteins outside the canonical replication complex (nsp7 to nsp16), including those in the structural and accessory cassette. Thus, while the immediate studies will focus on testing whether replicase proteins nsp8, nsp9, nsp13, and nsp15 regulate fidelity, it is exciting to consider the possibility that this virus-directed discovery approach will reveal novel interactions between multiple MHV proteins.

### Which mechanisms other than increased fidelity might account for MHV-ExoN(-) P250 nucleoside analog resistance?

Genomic mutations in RNA viruses are most commonly detrimental or lethal (8–12). One strategy to prevent extinction by mutational load is to increase replication fidelity, as discussed above. An alternative strategy is to increase mutational robustness. Mutational robustness describes the capacity of a virus to buffer the fitness effects of mutations. In the setting of low-fidelity replication, as in MHV-ExoN(-), increased mutational robustness could have provided a selective advantage (80–82). Selection for increased robustness could explain the ~90 synonymous changes in MHV-ExoN(-) P250. Synonymous changes can alter codons to reduce the probability of nonconservative amino acid changes (83). Alternatively, the increased population size of MHV-ExoN(-) P250 could promote robustness by a “safety-in-numbers” effect, allowing efficient purging of low-fitness mutants while maintaining population fitness (84). Large populations also increase the likelihood of coinfection, allowing complementation between viral genomes. Although increased replication conferred by mutations in spike did not alter 5-FU resistance (Fig. 5), results of a recent study performed with poliovirus suggest that mutagenized populations have elevated coinfection frequencies (85). Thus, complementation may contribute to MHV-ExoN(-) P250 nucleoside analogue resistance. Conflicting evidence exists regarding whether mutational robustness itself affects the sensitivity to RNA mutagens (83, 86, 87); nevertheless, the robustness of MHV-ExoN(-) P250 merits further investigation.

### Conclusions.

The proofreading activity of the nsp14 exoribonuclease is a critical determinant of CoV replication, fidelity, and fitness. We showed that CoVs have the capacity to compensate for loss of ExoN activity through a network of mutations in nsp12 and nsp14 and elsewhere in the genome. Thus, while nsp14-ExoN appears to play a dominant role in CoV replication fidelity, its activity is likely closely tied to a highly evolved network of proteins. The demonstrated coadaptation for replication, competitive fitness, and likely increased fidelity within MHV-ExoN(-) supports the hypothesis that these roles are linked functionally and evolutionarily. It will be interesting to test whether evolution in other cell types derived from different species or with different innate immune environments would result in similar adaptive strategies. Genetic and biochemical testing of the rich mutational resource revealed in MHV-ExoN(-) P250 will likely inform the design of countermeasures for endemic and emerging CoVs by defining novel common targets for stable virus attenuation or direct inhibition.

## MATERIALS AND METHODS

### Cell culture.

DBT-9 (delayed brain tumor, murine astrocytoma clone 9) cells were maintained as described previously (88). Baby hamster kidney (BHK) cells stably expressing the MHV-A59 receptor CEACAM1 (BHK-R; 15) were maintained under conditions of selection with 0.8 mg/ml of G418 (Mediatech) as described previously (88).

### Long-term passage of virus and stock generation.

The infectious cDNA clone for MHV-A59 and the recovery of MHV-ExoN(-) were described previously (14, 88). Long-term passage was initiated by infecting subconfluent monolayers of DBT-9 cells in 25-cm^2^ flasks with either wild-type MHV-A59 or MHV-ExoN(-) at an MOI of approximately 0.1 PFU/cell. One lineage of each virus was subjected to a total of 250 passages (P250). Supernatant was harvested at each passage and stored at −80°C. Total RNA was harvested for most passages using 1 ml of TRIzol reagent (Ambion) per 25-cm^2^ flask and stored at −80°C. Virus stocks of select intermediate passages were generated by infecting a subconfluent 150-cm^2^ flask of DBT-9 cells at an MOI of 0.01 PFU/cell. At approximately 24 hpi, the flask was frozen at −80°C and the supernatant was clarified by centrifugation at 4,000 × *g* (Sorvall RC-3B Plus; HA-6000A rotor) for 10 min at 4°C. The virus titer of each stock was determined by plaque assay using DBT-9 cells as described previously (14, 88). For plaque assays of viruses containing the spike protein from MHV-ExoN(-) P250, which does not form syncytia, plaques were visualized with neutral red (Sigma catalog no. N6264) (dilution at 1:10 in phosphate-buffered saline [PBS] containing calcium and magnesium). Neutral red was added 24 h after plating, and the reaction mixture was incubated for an additional 3 to 8 h before formaldehyde fixation. Plaque purification was performed by infecting DBT cells with serial dilutions of virus and overlaying the cultures with agar. Single plaques were isolated, resuspended in PBS containing calcium and magnesium, and inoculated onto fresh DBTs. This process was completed 3 times before experimental stocks were generated as described above.

### Sequencing of virus stocks.

Following P250, RNA was purified from the harvested TRIzol samples according to the manufacturer’s protocol and reverse transcribed (RT) using SuperScript III (Invitrogen) as described previously (14). Full-genome di-deoxy sequencing was performed for both WT-MHV P250 and MHV-ExoN(-) P250 using 12 overlapping amplicons approximately 3 kb in length. All coding regions were sequenced fully, and, of 31,409 nucleotides, >99% were sequenced for each virus [for WT-MHV P250, 21 to 31,279; for MHV-ExoN(-) P250, 21 to 31,275]. Two microliters of RT product was used for each PCR (16). Di-deoxy sequencing was performed by Genhunter Corporation (Nashville, TN) and Genewiz (South Plainfield, NJ). Sequence analysis was performed using MacVector version 14 (MacVector, Inc., Apex, NC) and the MHV-A59 infectious clone reference genome (GenBank accession number AY910861). The nucleotide sequences of the amplicon and sequencing primers are available upon request. Sequencing of nsp12 and nsp14 from intermediate passages was performed using TRIzol-purified RNA from infected monolayers and the primers listed below. Primers 6M1F (5′-TTTTGGCGAGATGGTAGC-3′) and 7M2R (5′-GGTAAGACAGTTTTAGGTGAG-3′) were used to generate a 3,425 nucleotide amplicon containing all of nsp12. Primers 7M3F (5′-ATGCTTACCAACTATGAGC-3′) and 8M3R (5′-CCGATTTGAATGGCGTAG-3′) were used to generate a 2,713 nucleotide amplicon containing all of nsp14. The PCR conditions for these reactions were the same as those used to generate the amplicons used for full-genome sequencing (16).

### Replication and RNA synthesis kinetics.

Viral replication kinetics in DBT-9 cells were determined at an MOI of 1 PFU/cell or an MOI of 0.01 PFU/cell as described previously (15). Supernatant (300 μl) was harvested at the indicated time points, and the virus titer was determined by plaque assay. The accumulation of genomic RNA at an MOI of 1 PFU/cell was measured by two-step real-time quantitative RT-PCR. Intracellular RNA was harvested using TRIzol and reverse transcribed using SuperScript III (Invitrogen). The levels of cDNA derived from intracellular positive-sense viral RNA were measured using primers directed to nsp10. Values were normalized to levels of the endogenous control glyceraldehyde-3-phosphate dehydrogenase (GAPDH). No mutations within the primer binding sites emerged in either P250 population. The primers and amplification conditions were the same as reported previously (15), except that the RT product was diluted 1:10 prior to use. Samples were plated in technical duplicate to minimize well-to-well variation. Data are presented as 2^−Δ*CT*^, where Δ*CT* denotes the threshold cycle (*C*_*T*_) value for the target (nsp10) minus the *C*_*T*_ value for the reference (GAPDH).

### Determination of specific infectivity.

Subconfluent monolayers of DBT-9 cells in 24-well plates were infected with the indicated virus at an MOI of 1 PFU/cell, and supernatant was harvested at 12 hpi. The levels of genomic RNA in supernatant were measured using one-step real-time quantitative RT-PCR (RT-qPCR) of TRIzol-extracted RNA as described previously (17). Briefly, genomic RNA was detected with a 5′ 6-carboxyfluorescein (FAM)-labeled and 3′ black hole quencher 1 (BHQ-1)-labeled probe targeting nsp2 (Biosearch Technologies, Petaluma, CA), and RNA copy numbers were calculated by reference to an RNA standard derived from the MHV A fragment. Samples were plated in technical duplicate to minimize well-to-well variation. Titers were determined by plaque assay in DBT-9 cells, and specific infectivity was calculated as PFU per supernatant genomic RNA copy.

### Nucleoside and base analogue sensitivity assays.

5-azacytidine (AZC), 5-fluorouracil (5-FU), and ribavirin (RBV) were purchased from Sigma (product numbers A2385, F6627, and R9644, respectively), and stock solutions were prepared in dimethyl sulfoxide (DMSO). CMeA (2′-C-methyladenosine) was received from Gilead Sciences (Foster City, CA). Sensitivity assays were performed as described previously (16), except that 24-well plates were used at an MOI of 0.01 PFU/cell. Supernatants were harvested at 24 hpi, and titers were determined by plaque assay.

### Phyre^2^ modeling of MHV-nsp14.

The MHV nsp14 structure was modeled with the Phyre^2^ online program (62) using nsp14 residues 3 to 519, corresponding to residues 6,056 to 6,573 of the ORF1ab polyprotein. The model was analyzed using the PyMOL Molecular Graphics System, Version 1.3 (Schrödinger, LLC).

### Generation of nsp12 and nsp14 swaps.

Viruses containing nsp12-P250 or nsp14-P250 or both were generated using the MHV-A59 reverse genetics system (88). RNA from the MHV-ExoN(-) P250 virus was reversed transcribed with SuperScript III (Invitrogen) and used to generate amplicons containing either nsp12 or nsp14. Each amplicon was flanked by 15 bp that overlapped an amplicon generated from the backbone plasmid. Amplicons were inserted into MHV-A59 fragments using an InFusion HD cloning kit (TaKaRa Bio USA, Inc., Mountain View, CA). nsp12 is split across MHV E and F, while nsp14 is contained within MHV F. Reaction mixtures contained 50 ng of vector, 200 ng of insertion, and 2 μl of enzyme and were incubated for 15 min at 50°C. Errors were corrected by site-directed mutagenesis using *Pfu* Turbo polymerase (Agilent, Santa Clara, CA). The nsp12/14-P250 swap was generated through restriction digestion of the individual swaps using BsmBI and StuI followed by gel purification and assembly using T4 DNA ligase (NEB, Ipswich, MA). Viable viruses were constructed and rescued as described previously (88).

### Competitive fitness assays.

Competitor viruses were competed with an MHV-ExoN(-) virus harboring 10 silent mutations in the probe-binding region within nsp2. Subconfluent DBT-9 monolayers in 24-well plates were coinfected at a total MOI of 0.01 PFU/cell with competitor and reference viruses at a 1:1 ratio and passaged 4 times. For each passage, supernatants were harvested at 24 h. RNA was extracted from 100 μl of supernatant using 900 μl of TRIzol reagent and PureLink RNA minikit columns (Thermo Scientific, Waltham, MA), and 150 μl of supernatant was used to infect fresh cells in a 24-well plate (total MOI estimated at 1 PFU/cell). The proportion of each virus was determined by real-time RT-qPCR from the infection supernatant using two TaqMan probes with different fluorescent dyes in separate reactions. Competitor viruses were detected with the same probe used in specific infectivity analyses (14). Reference viruses were detected by a probe targeting the same region but with 10 silent mutations (5′-TCCGAACTACTGCAACCCCAAGTG-3′) and labeled with 5′ quasar 670 and 3′ black hole quencher 2 (BHQ-2) (Biosearch Technologies, Petaluma, CA). RNA copy numbers were calculated by reference to an RNA standard generated by *in vitro* transcription of the corresponding MHV A fragment, and relative RNA abundances were calculated as ratios of competitor genomes to reference genomes.

### Statistical analysis.

GraphPad Prism 6 (La Jolla, CA) was used to perform statistical tests. Only the comparisons shown (with statistical significance indicated as "ns" [nonsignificance] or asterisk[s]) within each figure or described in each legend were performed. In many cases, the data were normalized to the results obtained with untreated controls. This was performed using GraphPad Prism 6. The number of replicate samples is denoted within each figure legend.

### Accession numbers.

Full-length genome sequences for WT-MHV P250 and MHV-ExoN(-) P250 have been deposited in GenBank (accession numbers MF618252 and MF618253, respectively).
