# Supplementary material for: Proofreading-Deficient Coronaviruses Adapt for Increased Fitness over Long-Term Passage without Reversion of Exoribonuclease-Inactivating Mutations
Source: mBio. 2017 Nov 7;8(6):e01503-17. doi: 10.1128/mBio.01503-17 (PMC5676041; doi:10.1128/mBio.01503-17)
Supplement: TABLE S1 [file mbo006173586st1.pdf]

Supplemental Table 1: Mutations in WT-MHV P250

Sequencing starts at nucleotide 21 and ends after nucleotide 31279.													
Double asterisk (**) denotes mixed nucleotides at approximately 50%-50% prevelance in the population.													
**Nucleotide Change in Genome**						**Codon**		**Amino Acid Change in Polyprotein**					
**Mutation**	**Position**	**nsp**	**Change**			**Original**	**New**	**Type**	**Original**	**Position: polyprotein (cleaved)**	**Mutation**	**Notes**	**nsp Boundary (nt)**
1	36	N/A	A	→	C	noncoding region							
2	645	1	G	→	A	GCC	ACC	Coding	Ala	146 (146)	Thr	Mixed; A>G	nsp1 (210-950)
3	1844	2	T	→	G	AGT	AGG	Coding	Ser	545 (298)	Arg		nsp2 (951-2705)
4**	1850	2	C	→	Y	CTC	CTY	Silent				Mixed; C≅T	
5	3139	3	A	→	G	GAA	GGA	Coding	Glu	977 (145)	Gly	Mixed; A>G	nsp3 (2706-8720)
6	5521	3	T	→	C	ATC	ACC	Coding	Ile	1771 (939)	Thr		
7	6257	3	T	→	G	AGT	AGG	Coding	Ser	2016 (1184)	Arg	Mixed; G>T	
8	6733	3	C	→	A	ACT	AAT	Coding	Thr	2175 (1343)	Asn	Mixed; A>C	
9	11448	6	G	→	A	GAT	AAT	Coding	Asp	3747 (111)	Asn	Mixed; A>G	nsp6 (11118-11978)
10	12416	8	T	→	C	GCT	GCC	Silent					nsp8 (12255-12836)
11	17871	13	G	→	T	AGT	ATT	Coding	Ser	5888 (504)	Ile		nsp13 (16361-18160)
12	20410	15	T	→	C	TCT	TCC	Silent					nsp15 (19724-20845)

**Nucleotide Change in Genome**						**Codon**		**Amino Acid Change in Accessory/Structural Protein**					
**Mutation**	**Position**	**Protein**	**Change**			**Original**	**New**	**Type**	**Original**	**Position**	**Mutation**	**Notes**	**Protein Boundary (nt)**
13	21971	ns2	A	→	T	CAA	CAT	Coding	Gln	67	His		ns2 (21771-22556)
Deletion: 22690-23878			The majority of was HE deleted. [HE: 22602-23921]										
14	24332	Spike	C	→	A	ACT	AAT	Coding	Thr	135	Asn	Mixed; A>C	Spike (23929-27903)
15	24673	Spike	A	→	C	ACA	CCA	Coding	Thr	249	Pro		
16	24843	Spike	C	→	T	GTC	GTT	Silent					
17	25476	Spike	T	→	A	AAT	AAA	Coding	Asn	516	Lys		
18	25749	Spike	C	→	A	ACC	ACA	Silent					
19	26630	Spike	G	→	T	CGT	CTT	Coding	Arg	901	Leu		
Deletion: 27924-28045			The majority of 4a was deleted. [4a: 27993-28052]										
20	28382	5a	C	→	T	CCA	CTA	Coding	Pro	3	Leu		5a (28375-28713)
21	28447	5a	A	→	C	ATT	CTT	Coding	Ile	25	Leu		
22	28928	E	C	→	A	CTG	ATG	Coding	Leu	75	Met	Mixed; A>C	E (28706-28957)
23	29650	M	C	→	T	ACC	ATC	Coding	Thr	228	Ile		M (28968-29654)
