# Supplementary material for: Proofreading-Deficient Coronaviruses Adapt for Increased Fitness over Long-Term Passage without Reversion of Exoribonuclease-Inactivating Mutations
Source: mBio. 2017 Nov 7;8(6):e01503-17. doi: 10.1128/mBio.01503-17 (PMC5676041; doi:10.1128/mBio.01503-17)
Supplement: TABLE S2 [file mbo006173586st2.pdf]

Supplemental Table 2: Mutations in MHV-ExoN(-) P250

Sequencing starts at nucleotide 21 and ends after nucleotide 31275.
Double asterisk (**) denotes mixed nucleotides at approximately 50%-50% prevelance in the population.

Nucleotide Change in Genome				Codon		Amino Acid Change in Polyprotein					
Mutation	Position	nsp	Change	Original	New	Type	Original	Position: polyprotein (cleaved)	Mutation	Notes	nsp Boundary (nt)
1	72	N/A	T → A	noncoding region							
2	227	1	A → C	AAA	AAC	Coding	Lys	6 (6)	Asn		
3	302	1	T → A	CCT	CCA	Silent					
4	371	1	G → A	TTG	TTA	Silent					
5	563	1	T → A	AAT	AAA	Coding	Asn	118 (118)	Lys		
6	645	1	G → A	GCC	ACC	Coding	Ala	146 (146)	Thr		nsp1 (210-950)
7	816	1	T → C	TCC	CCC	Coding	Ser	203 (203)	Pro		
8	839	1	T → C	GTT	GTC	Silent					
9	927	1	G → A	GCT	ACT	Coding	Ala	240 (240)	Thr		
10	1496	2	T → A	GGT	GGA	Silent					
11**	1623	2	A → W	ATG	WTG	Coding	Met	472 (225)	Xxx	Mixed; A≅T	
12	1927	2	T → C	GTA	GCA	Coding	Val	573 (326)	Ala		nsp2 (951-2705)
13	2520	2	G → A	GAT	AAT	Coding	Asp	771 (524)	Asn		
14	2696	2	T → C	TTT	TTC	Silent				Mixed; C>T	
15	2741	3	C → T	GTC	GTT	Silent					
16	3223	3	A → T	GAG	GTG	Coding	Glu	1005 (173)	Val		
17	3231	3	G → A	GCT	ACT	Coding	Ala	1008 (176)	Thr		
18	3371	3	T → A	TTT	TTA	Coding	Phe	1054 (222)	Leu		
19	3428	3	G → A	GCG	GCA	Silent					
20	3628	3	T → C	TTG	TCG	Coding	Leu	1140 (308)	Ser		
21	3697	3	A → T	AAG	ATG	Coding	Lys	1163 (331)	Met		
22	3872	3	T → C	TCT	TCC	Silent					
23	3903	3	T → A	TTG	ATG	Coding	Leu	1232 (400)	Met	Mixed; A>T	
24	3942	3	G → A	GTG	ATG	Coding	Val	1245 (413)	Met		
25	3974	3	G → A	ACG	ACA	Silent					
26	4280	3	T → A	GTT	GTA	Silent				Mixed; A>T	
27	4604	3	T → C	AGT	AGC	Silent				Mixed; C>T	
28	4853	3	C → T	GAC	GAT	Silent					
29	4880	3	T → C	GTT	GTC	Silent					
30	4994	3	T → C	TTT	TTC	Silent				Mixed; C>T	
31	5214	3	G → A	GAG	AAG	Coding	Glu	1669 (837)	Lys	Mixed; A>G	
32	5255	3	T → C	GAT	GAC	Silent					
33	5627	3	T → A	GTT	GTA	Silent					
34	5636	3	T → A	GTT	GTA	Silent				Mixed; A>T	
35	5951	3	T → C	TTT	TTC	Silent					nsp3 (2706-8720)
36	6119	3	G → A	TTG	TTA	Silent					
37	6151	3	C → T	GCT	GTT	Coding	Ala	1981 (1149)	Val		
38	6194	3	A → C	AAA	AAC	Coding	Lys	1995 (1163)	Asn		
39	6211	3	C → A	ACA	AAA	Coding	Thr	2001 (1169)	Lys		
40	6305	3	T → A	CTT	CTA	Silent					
41**	6420	3	C → Y	CCT	YCT	Coding	Pro	2071 (1239)	Xxx	Mixed; T≅C	
42	6489	3	A → G	AAG	GAG	Coding	Lys	2094 (1262)	Glu		
43	6516	3	A → G	AGT	GGT	Coding	Ser	2103 (1271)	Gly		
44	6520	3	T → A	GTG	GAG	Coding	Val	2104 (1272)	Glu		
45	6602	3	T → G	GTT	GTG	Silent					
46	6648	3	A → T	ACT	TCT	Coding	Thr	2147 (1315)	Ser		
47	6942	3	T → A	TTT	ATT	Coding	Phe	2245 (1413)	Ile	Mixed; A>G	
48	7113	3	G → A	GCT	ACT	Coding	Ala	2302 (1470)	Thr		
49	7381	3	T → C	ATT	ACT	Coding	Ile	2391 (1559)	Thr	Mixed; C>T	
50	7619	3	T → C	TGT	TGC	Silent				Mixed; C>T	
51	7748	3	T → C	GCT	GCC	Silent					
52	8099	3	C → T	GCC	GCT	Silent				Mixed; T>C	
53	8162	3	T → C	ACT	ACC	Silent				Mixed; C>T	
54	8642	3	C → A	GGC	GGA	Silent				Mixed; A>G	
55	8909	3	C → T	AAC	AAT	Silent				Mixed; T>C	
56**	9254	3	T → Y	TCT	TCY	Silent				Mixed; C≅T	
57	9287	3	T → C	TAT	TAC	Silent				Mixed; C>T	
58	9380	3	C → T	TGC	TGT	Silent					
59	9498	3	T → A	TTT	ATT	Coding	Phe	3097 (260)	Ile		nsp4 (8721-10208)

60	9887	4	A	→	T	GCA	GCT	Silent					
61	10010	4	T	→	A	TCT	TCA	Silent					
62	10145	4	T	→	C	CAT	CAC	Silent					
63	10188	4	G	→	A	GTT	ATT	Coding	Val	3327 (490)	Ile		
64	10382	5	T	→	C	CTT	CTC	Silent					nsp5 (10209-11117)
65	11710	6	T	→	A	TTC	TAC	Coding	Phe	3834 (198)	Tyr		nsp6 (11118-11978)
66**	12398	8	G	→	R	GAG	GAR	Silent				Mixed; A≅G	
67	12720	8	T	→	C	TTT	CTT	Coding	Phe	4171 (248)	Leu		nsp8 (12255-12836)
68	12794	8	T	→	A	ATT	ATA	Silent					
69	12830	8	T	→	A	GTT	GTA	Silent					
70	12973	9	G	→	A	TGT	TAT	Coding	Cys	4255 (46)	Tyr		nsp9 (12837-13166)
71	13331	10	T	→	C	ATT	ATC	Silent				Mixed; C>T	nsp10 (13167-13577)
72	13841	12	T	→	C	TTG	CTG	Silent					
73	13966	12	A	→	T	GCA	GCT	Silent					
74	14077	12	G	→	A	GAG	GAA	Silent				Mixed; A>G	
75	14290	12	T	→	C	TAT	TAC	Silent					
76	14320	12	T	→	C	TGT	TGC	Silent					
77	14439	12	T	→	C	ATG	ACG	Coding	Met	4744 (288)	Thr		
78	14703	12	T	→	C	CTT	CCT	Coding	Leu	4832 (376)	Pro		
79	15436	12	T	→	A	CGT	CGA	Silent					
80	15701	12	C	→	T	CAC	TAC	Coding	His	5165 (709)	Tyr		nsp12 (13601-16360)
81	15873	12	T	→	A	TTT	TAT	Coding	Phe	5222 (766)	Tyr		
82	15902	12	A	→	C	AGT	CGT	Coding	Ser	5232 (776)	Arg		
83	15952	12	G	→	A	GAG	GAA	Silent					
84	16017	12	T	→	A	ATG	AAG	Coding	Met	5270 (814)	Lys		
85	16150	12	T	→	A	CTT	CTA	Silent				Mixed; A>T	
86	16273	12	C	→	T	TAC	TAT	Silent					
87	16294	12	T	→	C	TGT	TGC	Silent				Mixed; C>T	
88**	16315	12	T	→	W	GAT	GAW	Coding	Asp	5369 (913)	Xxx	Mixed; C≅T	
89	16369	13	T	→	C	GGT	GGC	Silent				Mixed; C>T	
90	16459	13	T	→	C	CAT	CAC	Silent					
91	16756	13	G	→	A	TTG	TTA	Silent					
92	17083	13	A	→	G	GTA	GTG	Silent					
93	17104	13	C	→	T	AGC	AGT	Silent					nsp13 (16361-18160)
94	17362	13	T	→	A	CCT	CCA	Silent					
95	17746	13	T	→	C	GCT	GCC	Silent					
96	17758	13	T	→	C	AAT	AAC	Silent				Mixed; C>T	
97	17836	13	A	→	G	ATA	ATG	Coding	Ile	5876 (492)	Met		
98	18298	14	T	→	A	TCT	TCA	Silent					
	18426	14	A	→	C	GAT	GCT	Coding	Asp	6073 (89)	Ala	ExoN- (engineered)	
	18427	14	T	→	A	GAT	GAA	Coding					
	18432	14	A	→	C	GAA	GCA	Coding	Glu	6075 (91)	Ala		
	18433	14	A	→	T	GAA	GAT	Coding					
99	18544	14	T	→	A	GAT	GAA	Coding	Asp	6112 (128)	Glu		
100	18748	14	T	→	C	TAT	TAC	Silent					
101	18807	14	T	→	A	TTT	TAT	Coding	Phe	6200 (216)	Tyr		
102	18902	14	T	→	C	TAT	CAT	Coding	Tyr	6232 (248)	His		
103	18910	14	A	→	G	GGA	GGG	Silent					nsp14 (18161-19723)
104	18976	14	T	→	A	GAT	GAA	Coding	Asp	6256 (272)	Glu		
105	19173	14	C	→	T	GCC	GTC	Coding	Ala	6322 (338)	Val		
106	19282	14	T	→	A	GGT	GGA	Silent					
107	19333	14	T	→	C	GTT	GTC	Silent					
108	19396	14	T	→	C	AGT	AGC	Silent					
109	19577	14	T	→	A	TTA	ATA	Coding	Leu	6457 (473)	Ile		
110	19627	14	T	→	A	CTT	CTA	Silent					
111	20120	15	A	→	G	AAT	GAT	Coding	Asn	6638 (133)	Asp	Mixed; G>A	
112	20125	15	C	→	T	GGC	GGT	Silent					nsp15 (19724-20845)
113	20266	15	T	→	C	GAT	GAC	Silent					
114	21265	16	G	→	C	GGG	GGC	Silent					nsp16 (20846-21742)
115	21751	N/A	T	→	C	noncoding region							

Nucleotide Change in Genome						Codon		Amino Acid Change in Accessory/Structural Protein					
**Mutation**	**Position**	**Protein**	**Change**			**Original**	**New**	**Type**	**Original**	**Position**	**Mutation**	**Notes**	**Protein Boundary (nt)**
116	21816	ns2	G	→	A	GCC	ACC	Coding	Ala	16	Thr		
117	22137	ns2	T	→	A	TGG	AGG	Coding	Trp	123	Arg		ns2 (21771-22556)
118	22274	ns2	T	→	C	GGT	GGC	Silent				Mixed; C>T	

Deletion: 22285-23647			ns2 was truncated and the majority of was HE deleted. [HE: 22602-23921]										
119	23771	HE	A	→	T	HE is a non-functional gene with the MHV-A59 reverse genetics system.							HE (22602-23921)
120	23882	HE	T	→	A								
121	23887	HE	T	→	A								
122	24120	S	G	→	A	TTG	TTA	Silent					Spike (23929-27903)
123	24168	S	T	→	C	GCT	GCC	Silent					
124	24273	S	G	→	A	ACG	ACA	Silent					
125	24387	S	C	→	T	TGC	TGT	Silent					
126	24435	S	T	→	C	ACT	ACC	Silent					
127	24438	S	T	→	A	AAT	AAA	Coding	Asn	170	Lys		
128	24630	S	T	→	A	GAT	GAA	Coding	Asp	234	Glu		
129	24736	S	T	→	A	TTT	ATT	Coding	Phe	270	Ile		
130	24869	S	A	→	G	CAA	CGA	Coding	Gln	314	Arg		
131	24938	S	C	→	A	GCT	GAT	Coding	Ala	337	Asp		
132	25039	S	T	→	G	TTT	GTT	Coding	Phe	371	Val		
133	25116	S	C	→	T	CCC	CCT	Silent					
134	25233	S	T	→	A	AAT	AAA	Coding	Asn	435	Lys		
135	25436	S	T	→	C	GTG	GCG	Coding	Val	503	Ala		
136	25562	S	A	→	G	GAT	GGT	Coding	Asp	545	Gly		
137	25605	S	T	→	C	AAT	AAC	Silent					
138	25812	S	T	→	C	TAT	TAC	Silent					
139	25851	S	A	→	T	GCA	GCT	Silent					
140	25878	S	T	→	C	GCT	GCC	Silent					
141	26068	S	A	→	G	AGG	GGG	Coding	Arg	714	Gly	Furin site	
142	26520	S	T	→	A	AGT	AGA	Coding	Ser	864	Arg		
143	26618	S	G	→	A	GGT	GAT	Coding	Gly	897	Asp		
144	26782	S	G	→	A	GTT	ATT	Coding	Val	952	Ile		
145	27210	S	C	→	T	GGC	GGT	Silent					
146	27249	S	T	→	A	CCT	CCA	Silent				Mixed; A>T	
147	27261	S	T	→	C	TAT	TAC	Silent					
148	27294	S	T	→	C	TTT	TTC	Silent					
149	27366	S	T	→	C	TTT	TTC	Silent					
150	27566	S	T	→	C	ATT	ACT	Coding	Ile	1213	Thr		
151	27771	S	G	→	A	GTG	GTA	Silent					
152	28010	4a	A	→	G	CTA	CTG	Silent					4a (27993-28052)
153	28027	4a	T	→	A	CTG	CAG	Coding	Leu	12	Gln		
154	28075	4b	A	→	T	CTA	CTT	Silent					4b (28058-28378)
155	28124	4b	A	→	T	ATA	TTA	Coding	Ile	23	Leu		
156	28254	4b	T	→	A	CTT	CAT	Coding	Leu	66	His	Mixed; A>G	
157	28286	4b	T	→	A	TTT	ATT	Coding	Phe	77	Ile		
158	28500	5a	T	→	C	GGT	GGC	Silent					5a (28375-28713)
159	28652	5a	T	→	A	TTA	TAA	Coding	Leu	93	***		
160	28673	5a	T	→	A	TTT	TAT	Coding	Phe	100	Tyr		
161	28727	E	G	→	A	GAC	AAC	Coding	Asp	8	Asn		E (28706-28957)
162	28813	E	T	→	A	TCT	TCA	Silent					
163	28900	E	T	→	A	CTT	CTA	Silent				Mixed; A>T	
164	28991	M	A	→	T	CCA	CCT	Silent					M (28968-29654)
165	29298	M	A	→	C	AGG	CGG	Silent					
166	29428	M	A	→	T	CAC	CTC	Coding	His	154	Leu		
167	29508	M	T	→	C	TCA	CCA	Coding	Ser	181	Pro		
168	30275	N	T	→	A	TCT	ACT	Coding	Ser	203	Thr	Mixed; A>T	N (29669-31033)
169	30329	N	A	→	T	AAC	TAC	Coding	Asn	221	Tyr	Mixed; T>A	
170	30469	N/A	T	→	A	ATT	ATA	Silent	noncoding region				
171	30955	N/A	A	→	G	CCA	CCG	Silent					
