# Supplementary figures and images for: Proofreading-Deficient Coronaviruses Adapt for Increased Fitness over Long-Term Passage without Reversion of Exoribonuclease-Inactivating Mutations
Source: mBio. 2017 Nov 7;8(6):e01503-17. doi: 10.1128/mBio.01503-17 (PMC5676041; doi:10.1128/mBio.01503-17)

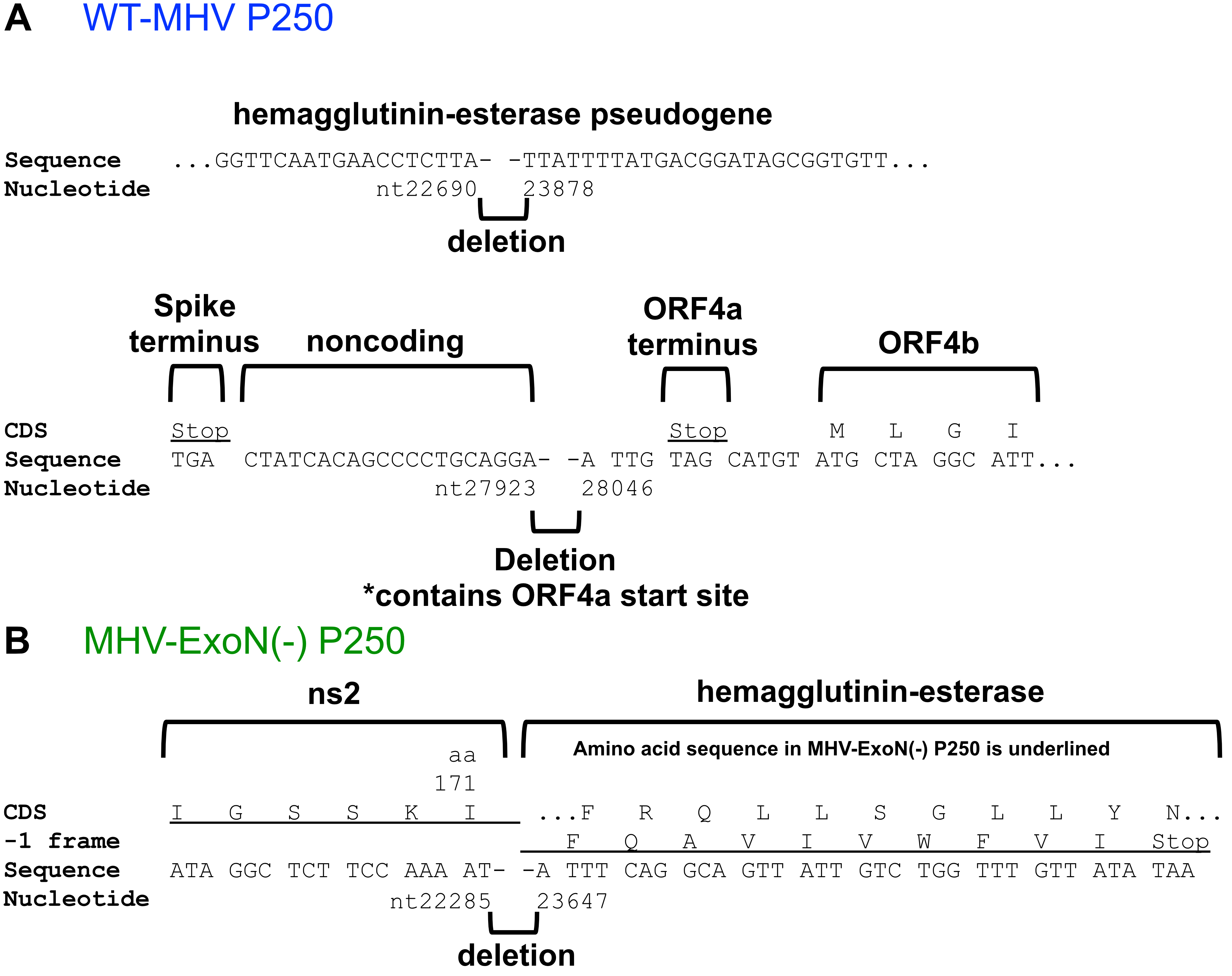

Supplement: FIG S1 [file mbo006173586sf1.tif]
